# The Inner Workings of the Outer Surface: Skin and Gill Microbiota as Indicators of Changing Gut Health in Yellowtail Kingfish

**DOI:** 10.3389/fmicb.2017.02664

**Published:** 2018-01-15

**Authors:** Thibault P. R. A. Legrand, Sarah R. Catalano, Melissa L. Wos-Oxley, Fran Stephens, Matt Landos, Matthew S. Bansemer, David A. J. Stone, Jian G. Qin, Andrew P. A. Oxley

**Affiliations:** ^1^Aquatic Sciences Centre, South Australian Research and Development Institute, West Beach, SA, Australia; ^2^School of Biological Sciences, Flinders University, Adelaide, SA, Australia; ^3^Research Group Microbial Interactions and Processes, Helmholtz Centre for Infection Research, Braunschweig, Germany; ^4^South Australian Museum, Adelaide, SA, Australia; ^5^Department of Fisheries, South Perth, WA, Australia; ^6^Future Fisheries Veterinary Service Pty Ltd., East Ballina, NSW, Australia

**Keywords:** skin, gills, mucosal microbiome, enteritis, aquaculture, *Seriola lalandi*, 16S rRNA

## Abstract

The mucosal surfaces and associated microbiota of fish are an important primary barrier and provide the first line of defense against potential pathogens. An understanding of the skin and gill microbial assemblages and the factors which drive their composition may provide useful insights into the broad dynamics of fish host–microbial relationships, and may reveal underlying changes in health status. This is particularly pertinent to cultivated systems whereby various stressors may led to conditions (like enteritis) which impinge on productivity. As an economically important species, we assessed whether the outer-surface bacterial communities reflect a change in gut health status of cultivated Yellowtail Kingfish (*Seriola lalandi*). Active bacterial assemblages were surveyed from RNA extracts from swabs of the skin and gills by constructing Illumina 16S rRNA gene amplicon libraries. Proteobacteria and Bacteroidetes were predominant in both the skin and gills, with enrichment of key β-proteobacteria in the gills (Nitrosomonadales and Ferrovales). Fish exhibiting early stage chronic lymphocytic enteritis comprised markedly different global bacterial assemblages compared to those deemed healthy and exhibiting late stages of the disease. This corresponded to an overall loss of diversity and enrichment of Proteobacteria and Actinobacteria, particularly in the gills. In contrast, bacterial assemblages of fish with late stage enteritis were generally similar to those of healthy individuals, though with some distinct taxa. In conclusion, gut health status is an important factor which defines the skin and gill bacterial assemblages of fish and likely reflects changes in immune states and barrier systems during the early onset of conditions like enteritis. This study represents the first to investigate the microbiota of the outer mucosal surfaces of fish in response to underlying chronic gut enteritis, revealing potential biomarkers for assessing fish health in commercial aquaculture systems.

## Introduction

The outermost surfaces of animals represent an important primary barrier, particularly in fish where they share an intimate relationship with their watery surroundings. Like mammals, the outer surfaces of fish play critical protective, regulatory and sensory roles, acting as the first line of defense (Marshall and Bellamy, 2010; Peatman et al., 2015). However, as an added feature, the epithelia of the skin and gills are coated in a secretion of mucus that is continuously replaced, and comprises immunologically active molecules that arise from the underlying lymphoid tissues which parallel those of the gut, hindering opportunistic pathogens (Magnadottir, 2010; Esteban, 2012; Rakers et al., 2013; Xu et al., 2013; Peatman et al., 2015; Derome et al., 2016). This mucus layer is also host to an array of indigenous microbiota in which bacteria are predominant (Esteban, 2012; Llewellyn et al., 2014; Merrifield and Rodiles, 2015; Lokesh and Kiron, 2016). Among other functions, these communities facilitate the development and homeostasis of host immunity (Sellon et al., 1998; Cebra, 1999; Lee and Mazmanian, 2010), recycle and remove waste products (van Kessel et al., 2016), and provide colonization resistance by competing for space and nutrients (Balcazar et al., 2006). Factors such as diet, salinity, seasonality, stress, and the environment are known to influence these mucosal-associated communities (Boutin et al., 2013a,b; Landeira-Dabarca et al., 2013; Hess et al., 2015; Larsen et al., 2015; Lokesh and Kiron, 2016) where dysbioses may enhance susceptibility to disease (Llewellyn et al., 2014; Hess et al., 2015). Balanced and complex interplays within the mucus layer are thus key for disease resistance (Jani and Briggs, 2014; Lowrey et al., 2015) and are pivotal for supporting health and fitness (Gómez and Balcázar, 2008).

The role of the mucus layer and its associated microbiota is particularly relevant to cultivated fish species, especially given that fish are often stocked at higher densities than found in the wild, which elevates stress and vulnerability to infectious agents and thus hinders the full success of an aquaculture enterprise (Jurado et al., 2015). Within the Southern Hemisphere, Yellowtail Kingfish (YTK, *Seriola lalandi*) is one such commercial species, where its successful cultivation is impeded by a variety of diseases and syndromes. In particular, diseases of the gastrointestinal tract like enteritis are especially problematic, where symptomatic features required for diagnosis often occur at the later (chronic) stages, when often it is too late for therapeutic intervention (Sheppard, 2005; Bansemer et al., 2015). Non-invasive approaches for early detection are thus highly needed. Biomarkers, including the profiling of biochemical and immunological features and global changes in the commensal microbiota have been proposed in this regard (Llewellyn et al., 2014; Brinchmann, 2016). Cataloging the key community members associated with healthy individuals and then understanding how shifts in microbial community composition drive or become a sign of disease onset, are particularly important. Next generation sequencing (NGS) technologies now allow the deep-surveillance of microbial communities including the uncultivable members (Federici et al., 2015), and have been applied to YTK (Ramirez and Romero, 2017) and other commercially relevant species like Atlantic Salmon (Llewellyn et al., 2016) where they may be used as an additional tool for the management of fish health.

Here we investigate whether the outer-surface bacterial communities reflect a change in gut health status of YTK and seek for biomarkers that may be used as early warning signs for underlying chronic conditions. A non-invasive sampling approach by swabbing the outer mucosal surfaces was used to characterize the skin and gill bacterial assemblages of healthy YTK and those exhibiting signs of chronic lymphocytic enteritis presenting with early and late stages of this condition. Active and likely resident community members of these niches were profiled from RNA samples by Illumina deep-sequencing of the hypervariable V1–V2 region of the 16S rRNA gene, with accompanying histological sections. In addition, seawater samples collected adjacent to the sea cages provided a catalog of the global bacterial assemblages of the surrounding environment. This study represents the first to investigate whether the onset of chronic gut enteritis is reflected in the outer-surfaces (mucosal-barriers) of fish, where such knowledge could be used to detect early signs of a potential shift in health status non-invasively, and thereby prompt more rapid management responses.

## Materials and Methods

### Experimental Design and Sampling Strategy

**FIGURE 1:**
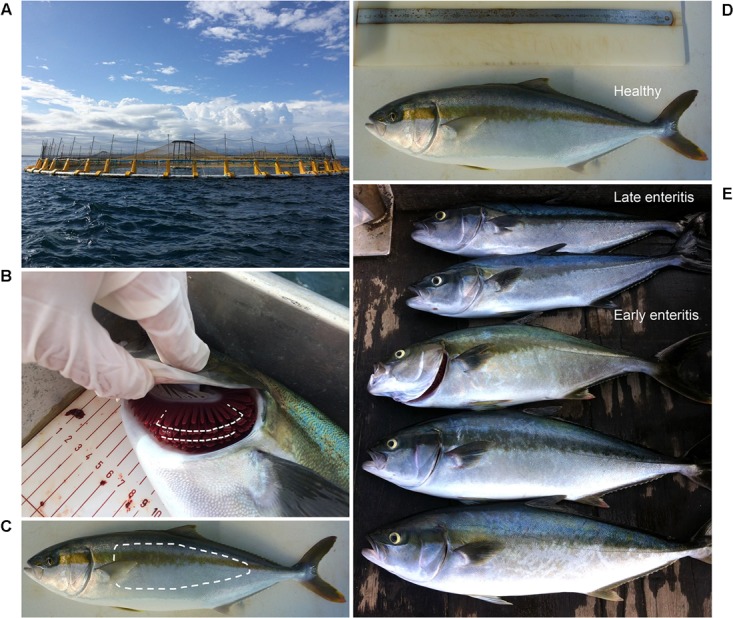
Study site and sampling approach. Farmed Yellowtail Kingfish (YTK, *Seriola lalandi*) were obtained from **(A)** sea cages from a commercial enterprise from temperate waters in southern Australia, where swab samples were taken from **(B)** gills and **(C)** skin within the regions denoted by dashed lines from **(D)** healthy individuals, and those with **(E)** early and late stages of enteritis.

A total of 36 fish were sampled under the auspices of a commercial aquaculture enterprise from temperate waters of southern Australia according to industry best practice veterinary care and included 12 from a control sea cage where no obvious signs of infection or a health problem were observed in the population prior to sampling (“healthy” group, sea cage A) and 24 from a sea cage containing mixed populations of individuals displaying early and late stages of enteritis (*n* = 12/group, sea cage B) (**Table 1**), as confirmed earlier by necropsy and histopathological assessment. These sea cages were located in the same geographical region (separated by a distance of <7 km), and comprised the same hatchery stock, where fish were fed the same diet and were transferred at similar sizes to the sea cages prior to sampling (**Figure 1**). Three wild-caught YTK (also from southern Australian waters, though distantly located to the farmed site) as well as two 1-L water samples (collected adjacent to each of the sea cages) were also obtained for analysis. Swabs of the skin and gills (**Figures 1B,C**) were promptly collected, stabilized in RNAlater^TM^ (Ambion, Austin, TX, United States) and stored at 4°C alongside the seawater samples before being transferred to -20°C until downstream RNA extraction. In addition, samples of the skin and gills were also collected for histopathological analysis following anesthetization in approximately 80 L of surrounding seawater comprising 20 mg L^-1^ AQUI-S (AQUI-S New Zealand Ltd.) solution as described previously (Bansemer et al., 2015). For this, a 1 cm^3^ section of the skin was collected along the lateral line at the same location for each fish, and for the gills, a section of the filaments was sampled from the second gill arch. Tissue samples were placed immediately in filter-sterilized seawater comprising 10% seawater formalin and stored at room temperature prior to analysis.

**Table 1:** Summary of the experiment, fish growth characteristics, and histopathological analyses.

	YTK group			
	
Factors	Wild-caught	Healthy	Early enteritis	Late enteritis
No. fish	3	12	12	12
Cage ID	–	A	B	B
Fork length (cm)	67.7 ± 6.3	61.4 ± 3.1	59.7 ± 2.5	49.3 ± 3.2
Weight (kg)	3.9 ± 1.1	3.4 ± 0.6	3.0 ± 0.3	1.4 ± 0.2
Fulton’s condition factor (K)	1.2 ± 0.1	1.5 ± 0.1	1.4 ± 0.2	1.2 ± 0.2
Skin thickness (μm)^a^	70.2 ± 12.9	67.8 ± 12.3	57.3 ± 14.0	49.3 ± 12.0
Skin thickness (no. cells)^a^	9.3 ± 1.9	9.8 ± 1.7	8.5 ± 1.2	6.8 ± 1.7
Total no. goblet cells^a^	4.5 ± 0.6	5.5 ± 1.6	3.3 ± 1.2	2.2 ± 1.1
No. acidic goblet cells^a^	3.9 ± 0.3	4.0 ± 1.2	2.3 ± 1.1	1.3 ± 0.7
No. neutral goblet cells^a^	0.5 ± 0.8	1.5 ± 0.9	1.0 ± 0.5	0.9 ± 0.8
Blood fluke viable egg count^b^	0 (0–1)	1.5 (0–39)	0 (0–40)	9.5 (0–60)
Epitheliocystis count^b^	1 (0–1)	0 (0–1)	0 (0–3)	0 (0–7)


*Means and standard deviations are presented, except for blood fluke viable egg and epitheliocystis counts where median and range are given. ^a^Per each 100 μm section; ^b^per primary lamellae.*

### Histological Evaluation

General tissue features (e.g., signs of inflammation and parasites, particularly blood fluke eggs and epitheliocystis) as well as the numbers of acidic mucus containing goblet cells were evaluated from formalin fixed hematoxylin and eosin and periodic acid-Schiff-Alcian Blue stained skin and gill tissue samples. Histological evaluations were conducted through the Department of Agriculture and Food Western Australian (DAFWA) Diagnostic and Laboratory Services (DDLS) – Animal Pathology Services and Department of Fisheries.

### Nucleic Acid Extraction, PCR Amplification, and Illumina Sequencing

To capture the active and thus likely resident community members of the YTK mucosa, RNA from stabilized skin and gill swabs was extracted by placing the tip of each swab into a lysing matrix B tube (MP Biomedicals) containing 1 mL of ice-cold RLT buffer supplemented with 1% β-mercaptoethanol v/v. Samples were disrupted via bead-beating using the FastPrep-24^TM^ 5G instrument (MP Biomedicals) at an intensity of 5.5 for 45 s, placed on ice for 3 min and then disrupted a second time using the same settings. The disrupted samples were subsequently centrifuged at 14,000 × *g* for 10 min at 4°C and the RNA extracted from the supernatant using the RNeasy mini kit (Qiagen) following the manufacturer’s instructions. To remove any contaminating gDNA, DNase treatment was performed for all samples using the Turbo DNA-free^TM^ kit (Life Technologies). Purified RNA was subsequently converted to cDNA using the Superscript^TM^ III First Strand Synthesis System (Life Technologies) according to the manufacturer’s instructions. In addition, to evaluate the contribution of the surrounding environmental microbial consortia on the YTK microbiome, DNA was extracted from the seawater samples following filtration onto 0.22 μM Nalgene^TM^ Rapid-Flow^TM^ filters (Thermo Fisher Scientific) using the FastDNA^TM^ Spin Kit for Soil (MP Biomedicals) according to the manufacturer’s instructions. All samples were concentrated by ethanol precipitation using standard procedures, quantified using the NanoDrop 2000 spectrophotometer and stored at -20°C prior to downstream library preparation.

The V1–V2 hypervariable region of the 16S rRNA gene was subsequently amplified from the cDNA and DNA extracts using a multi-step approach, with pre-enrichment using universal eubacterial primers 27F and 338R as described previously (Camarinha-Silva et al., 2014; Chaves-Moreno et al., 2015). More specifically, for library generation, 2 μL of cDNA and 5 μL of each DNA sample were first subjected to 20 cycles of PCR, whereby 1 μL of this mixture in the first round was used as template in a further 15 cycles of PCR for incorporating individual 6 nt barcodes and Illumina specific adaptors. One microliter of this reaction served as a template in a final 10-cycle PCR round for incorporating the Illumina multiplexing sequencing and index primers. Samples were visualized via gel electrophoresis and products of the expected size (∼438 bp) were purified using Agencourt AMPure XP beads (Beckman Coulter). Samples were quantified using the Quant-iT^TM^ Picogreen^®^ dsDNA kit (Life Technologies) following the manufacturer’s instructions and pooled in equimolar ratios before being sequenced on the MiSeq platform (Illumina, San Diego, CA, United States) using 250 nt paired-end sequencing chemistry through the Australian Genome Research Facility (AGRF, North Melbourne, VIC, Australia). Amplicons generated from gDNA from a single bacterial species (*Lactobacillus reuteri*) were sequenced alongside the samples as controls.

### Bioinformatics and Statistical Analysis

The sequenced samples returned ∼7.5 million raw reads. Reads were paired using PEAR version 0.9.5 (Zhang et al., 2014), where primers were identified and removed. The 3,766,157 paired-end reads were quality filtered, with removal of low-quality reads, full-length duplicate sequences (after being counted), and singleton sequences using Quantitative Insights into Microbial Ecology (QIIME 1.8; Caporaso et al., 2010), USEARCH (version 8.0.1623; Edgar, 2010), and UPARSE software (Edgar, 2013). Reads were mapped to operational taxonomic units (OTUs) using a minimum identity of 97%, removing putative chimeras using the RDP-gold database as a reference (Cole et al., 2014).

A total of 3,179,966 high-quality, paired-end reads were clustered into 14,712 OTUs. These OTUs were further filtered as conducted previously (Zhang et al., 2016) where only those that contributed to >0.01% of the host-associated dataset (78 samples) or >0.01% of the seawater samples were used, leaving a total of 930 OTUs and their respective abundance for the downstream analysis. Each of the YTK samples comprised a mean of 35,017 reads (ranging 17,155–58,439). Each of the seawater samples comprised 108,144 and 118,372 reads. Rarefaction curves were used to inspect (retrospectively) sampling depth (Supplementary Figure S1). Interrogation of the resultant OTUs was conducted using the Seqmatch function of the RDP database (Wang et al., 2007) as well as SILVA (Quast et al., 2013), whereby lineages based on the SILVA taxonomy and the best hit from RDP were assigned for each OTU (Supplementary Datasheet 1).

The data matrix comprising the percent standardized abundances of 930 OTUs across all 80 samples was first used to explore for patterns across the global bacterial communities, where samples were ordinated using non-metric multidimensional scaling (nMDS) with 50 random restarts (Clarke and Warwick, 2001) following construction of a sample-similarity matrix using the Bray–Curtis algorithm (Bray and Curtis, 1957). Significant differences between *a priori* predefined groups of samples (skin vs gill; and healthy vs early enteritis vs late enteritis) were evaluated using both two-way and one-way permutational multivariate analysis of variance (PERMANOVA), allowing for type III (partial) sums of squares, fixed effects of sum to 0 for mixed terms, and exact *p*-values generated using unrestricted permutation of raw data (Anderson, 2001). In addition, analysis of similarity (ANOSIM), where after 9999 permutations the accompanying R statistic measured the degree of separation between groups (Clarke and Warwick, 2001). Like with the nMDS analysis, PERMANOVA and ANOSIM used the Bray–Curtis similarity matrix. Groups of samples were considered significantly different if the *p*-value falls <0.05. These multivariate analyses were performed in PRIMER (version 7.0.11) PRIMER-E, Plymouth Marine Laboratory, United Kingdom (Clarke and Warwick, 2001). When significant differences were observed between groups, differential abundance analysis (taking into account effect size) was used to seek for those OTUs that contribute mostly to the observed differences using STAMP version 2.1.3 (Parks et al., 2014). First, those 764 OTUs only found in the healthy skin and healthy gill samples were compared using the Welch’s *t*-test (two-sided) that allows for unequal variances between the groups, using the Benjamini–Hochberg FDR correction to correct *p*-values and allow for multiple testing. Then, those 785 OTUs of the sea caged fish skin and 817 OTUs of the sea caged fish gills were compared using the Kruskal–Wallis *H*-test with the Games–Howell *post hoc* test (designed for unequal variances), also using the Benjamini–Hochberg FDR correction. OTUs were considered to be significantly different if the corrected *p*-value (*q*-value) falls <0.05.

Conventional measures of species diversity, richness, and evenness were calculated using algorithms for total OTUs (S), Shannon diversity (*H*′), Simpson (1 - λ), and Pielou’s evenness (*J*′), while taxonomic diversity was calculated using algorithms for taxonomic distinctness (TD): average TD (delta+) and variation in TD (lambda+) (Clarke and Warwick, 2001). Delta+ represents the average taxonomic distance between all pairs of species within each sample, and thus is a summary of average taxonomic breadth of each sample, while lambda+ reports how consistent each level of organization within the Linnaean classification is represented (Warwick and Clarke, 1995; Pienkowski et al., 1998). The expected delta+ values were calculated by sampling different numbers of OTUs (from 5 to 600 in increments of 10) from the master list of 930 OTUs, with 999 iterations. In addition, k-dominance plots and rarefaction curves were constructed using PRIMER (version 7.0.11). These univariate indicators of diversity [S, *H*′, 1 - λ, *J*′, average TD, variation TD, Proteobacteria:Bacteroidetes (P:B) ratio] as well as the univariate measures from histology (skin thickness and numbers of goblet cells) and growth parameters (fork length, weight, and Fulton’s condition factor) were compared between *a priori* groups of samples (wild-caught, healthy, early and late enteritis) using either the unpaired Welch’s *t*-test (not assuming equal variance) or ANOVA, after first subjecting each variable of interest to a normality test using both the D’Agostino and Pearson omnibus and the Shapiro–Wilk algorithms (Prism version 7.01, GraphPad Software Inc.). Variables were considered to be significantly different if the *p*-value falls <0.05. For the further presentation of data: Venn diagrams were constructed in the web tools available from Bioinformatics and Evolutionary Genomics, University of Gent, Belgium^1^; ternary plots were constructed in R using ggtern; and heatmaps were generated in Prism version 7.01 (GraphPad). The OTU tables on which the analyses were derived are presented in Supplementary Datasheet 1.

### Data Deposition

The OTU tables on which the analyses were derived are presented in Supplementary Datasheet 1. Sequences from individual samples were deposited within NCBI SRA repository under study SUB2879742; accession numbers SAMN07426671–SAMN07426750.

## Results

To explore bacterial community dynamics in the skin and gills of YTK and biomarkers of underlying gastrointestinal disorders (e.g., chronic lymphocytic enteritis), groups of fish of three different health states (*n* = 12 fish) were compared (**Table 1** and **Figure 1**). Despite the same starting status, a general decline in the mean fork length and weight was observed across the groups from healthy to early to late stage enteritis, where the mean weights were 3.4, 3.0, and 1.4 kg, and the fork length was 61.4, 59.7, and 49.3 cm, respectively (**Table 1** and Supplementary Figures S2A,B). However, only a significant difference was observed between the late stage enteritis and healthy/early enteritis groups. In addition, Fulton’s body condition factor was significantly lower in the late stage enteritis fish compared to the healthy fish, indicating overall poorer condition and not just smaller sizes (Supplementary Figure S2C). This was also evident for the reduction of skin thickness with health state from 67.8 to 49.3 μm (**Table 1** and Supplementary Figure S3). No inflammation or lesions of the skin were apparent. Furthermore, the total numbers of goblet cells and those which were classified as being acidic decreased significantly with fish health, with relative mean values ranging from 5.5 to 2.2 for total goblet cells and 4.0 to 1.3 for those that were acidic (**Table 1** and Supplementary Figure S4). Counts of viable blood fluke eggs and epitheliocystis from the gills were highly variable between fish from the same group where in most cases the counts were negligible (Supplementary Figure S5), although many of the gills had indications of granulomas where fluke eggs had been present. Taken together, this indicates that the underlying disease process of chronic lymphocytic enteritis led to widespread changes in growth, condition and the epithelium barrier system.

**FIGURE 2:**
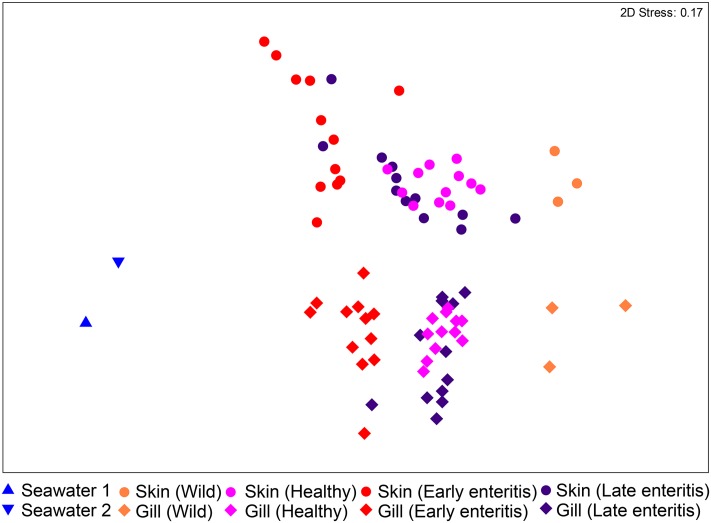
Ordination plot representing the differences in the global bacterial community structures of the skin and gills of 39 YTK (including wild-caught, healthy farmed YTK, and farmed YTK with early and late stage enteritis) and two seawater samples collected from the sea cages holding the healthy farmed group (seawater 1) and the early and late stage enteritis groups (seawater 2), as assessed by non-metric multidimensional scaling (nMDS) using Bray–Curtis dissimilarity.

The bacterial communities of the skin and gills were surveyed from swabs, as well as two seawater samples collected from the sea cages as controls. Among the derived 930 OTUs, 377 were unique to the skin and gills, 77 to seawater, and 476 that were shared between seawater and the skin/gills (20 skin/seawater; 61 gills/seawater; 395 skin/gills/seawater). Ordination of the samples revealed that despite the large number of shared OTUs, the skin and gill samples clustered independently to seawater (**Figure 2**). The wild-caught fish (though only three individuals) separated from the farmed sea cage fish, where the skin and gills clustered independently based on health status. This observation was confirmed by the two-way PERMANOVA, which crossed swabbed region with health, stating that there were highly significant differences between regions and globally between health states (pseudo-*F* = 22.241, *p*-value = 0.0001, pseudo-*F* = 10.373, *p*-value = 0.0001, respectively) (Supplementary Table S1). Despite a similar trend in changes across health states in both regions (**Figure 2**), there was a significant interaction between region and health status (pseudo-*F* = 3.6791, *p*-value = 0.0001), indicating skin and gill specific changes. Noteworthy, in both the skin and gills the early enteritis group separated out further from both healthy and late stage enteritis groups, with a less disparate (though statistically different) separation between the healthy and late enteritis states.

**FIGURE 3:**
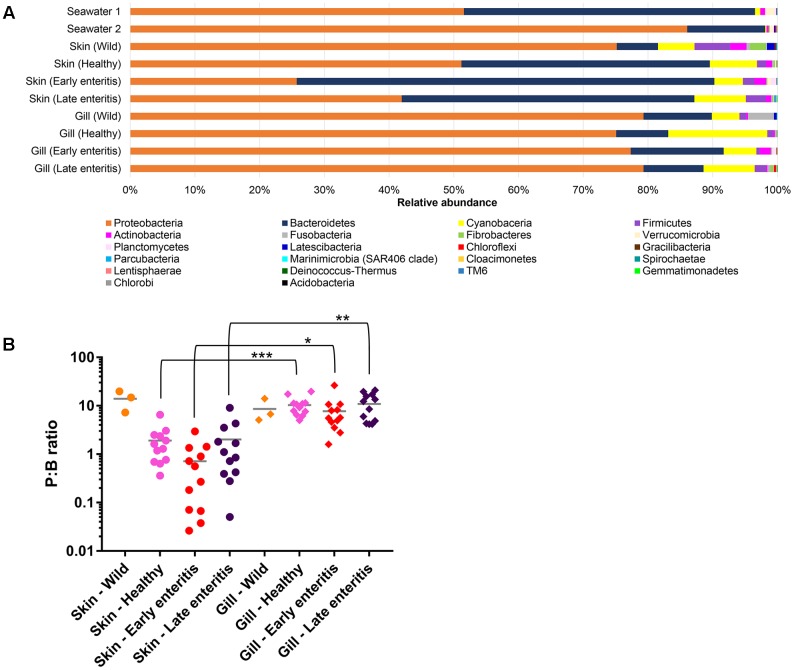
**(A)** Mean relative abundances of bacterial phyla associated with the skin and gills of wild-caught YTK, healthy farmed YTK, farmed YTK with early and late stage enteritis and seawater samples collected from the sea cages holding the healthy farmed group (seawater 1) and the early and late stage enteritis groups (seawater 2); **(B)** Ratio of the relative abundances of the Proteobacteria to Bacteroidetes (P:B ratio) from the skin and gills of the YTK groups, where the mean and standard deviation are presented. Differences were evaluated between the skin and gills within each group using Welch’s *t*-test, with the levels of statistical significance between groups denoted by asterisks, with alpha set at 0.05.

Overall, OTUs represented 22 phyla, 50 classes, 109 orders, 189 families, and 380 genera, where the two phyla Proteobacteria and Bacteroidetes comprised >80% of the OTU abundance (**Figure 3A**). Variations in the proportions of these phyla (as a P:B ratio) may be a useful metric to assess changes between groups of interest (**Figure 3B**). The 15 dominant YTK-associated OTUs collectively accounted for >50% of the total standardized sequence reads. These represent mostly unclassified and/or uncultured taxa related to *Lutibacter* sp. (OTU 3, 13.1%), *Nitrosomonas* sp. (OTU 6, 6.6%), *Polaribacter* sp. (OTU 8, 6.0%), unclassified Neisseriaceae sp. (OTU 10, 4.8%), *Ferrovum* spp. (OTU 11, 4.2% and OTU 18, 4.0%), unclassified β-proteobacteria SC-I-84 (OTU 15, 3.5 %), chloroplast (OTU 23, 2.2%), *Polaribacter* sp. (OTU 24, 1.9%), *Pelagibacter* sp. (OTU 9, 1.9%), chloroplast (OTU 26, 1.8%), *Pseudoalteromonas* sp. (OTU 38, 1.4%), *Roseovarius* sp. (OTU 32, 1.4%), unclassified Nitrosomonadaceae sp. (OTU 27, 1.2%), and *Ascidiaceihabitans* sp. (OTU 397, 1.1%) (Supplementary Datasheet 1).

### Defining the Skin- and Gill-Associated Bacterial Assemblages of Healthy YTK

**FIGURE 4:**
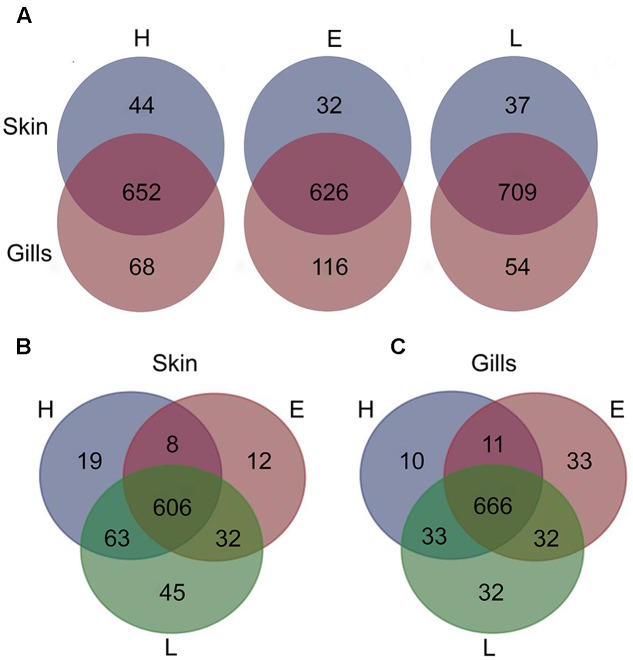
Venn diagrams showing the distribution of unique and shared OTUs in the skin and gills of farmed YTK of varying health status (H, healthy; E, early enteritis; L, late enteritis) where each plot represents differences in **(A)** skin and gills, **(B)** skin, and **(C)** gills.

**FIGURE 5:**
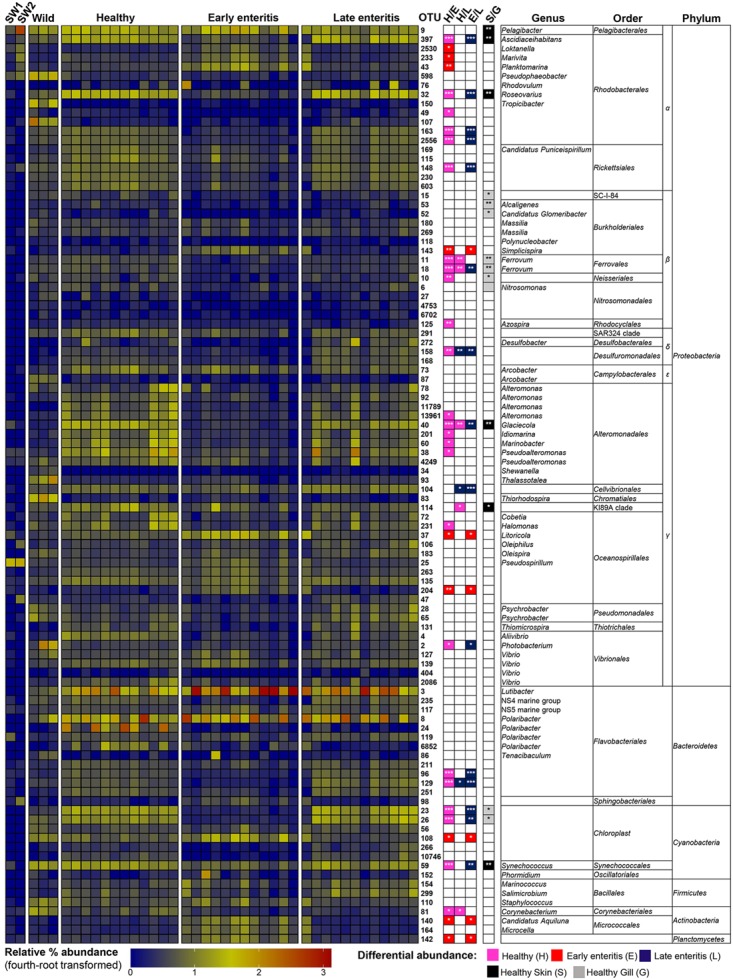
Heatmap showing the relative abundances of the OTUs from the skin of wild-caught YTK, healthy farmed YTK, and farmed YTK with early and late stage enteritis, and from seawater (SW1—healthy sea cage; SW2—early/late enteritis sea cage). The top 100 most abundant OTUs are plotted with color intensity representing fourth-root transformed values. The taxonomy of the OTUs (genus, order, and phylum) is depicted on the right. Colored squares with asterisks on the right of the plot indicate those OTUs that were considered statistically significant between different states of health and between gills/skin, where the particular color denotes in which status/region it was more abundant. Differential abundance analysis was performed between different states of health using the Kruskal–Wallis test with the Games–Howell *post hoc* test, or between healthy skin and gills using the Welch’s *t*-test. In both cases, *p*-values were corrected for using the Benjamini–Hochberg multiple comparison test, where alpha was set at 0.05.

**FIGURE 6:**
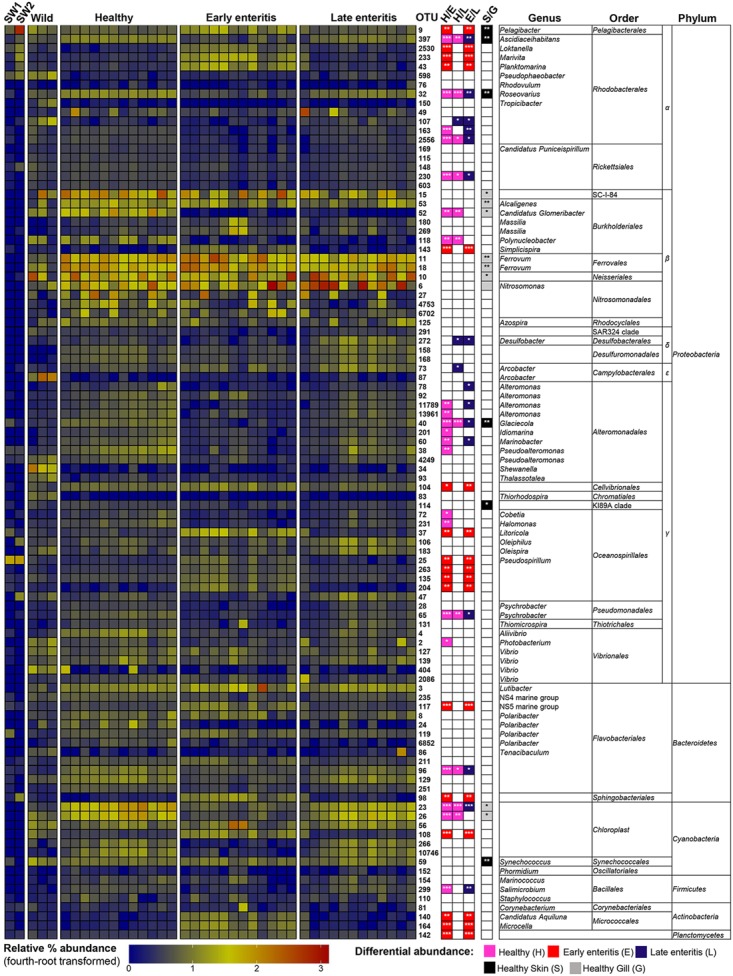
Heatmap showing the relative abundances of the OTUs from the gills of wild-caught YTK, healthy farmed YTK, and farmed YTK with early and late stage enteritis, and from seawater (SW1—healthy sea cage; SW2—early/late enteritis sea cage). The top 100 most abundant OTUs are plotted with color intensity representing fourth-root transformed values. The taxonomy of the OTUs (genus, order, and phylum) is depicted on the right. Colored squares with asterisks on the right of the plot indicate those OTUs that were considered statistically significant between different states of health and between gills/skin, where the particular color denotes in which status/region it was more abundant. Differential abundance analysis was performed between different states of health using the Kruskal–Wallis test with the Games–Howell *post hoc* test, or between healthy skin and gills using the Welch’s *t*-test. In both cases, *p*-values were corrected for using the Benjamini–Hochberg multiple comparison test, where alpha was set at 0.05.

**FIGURE 7:**
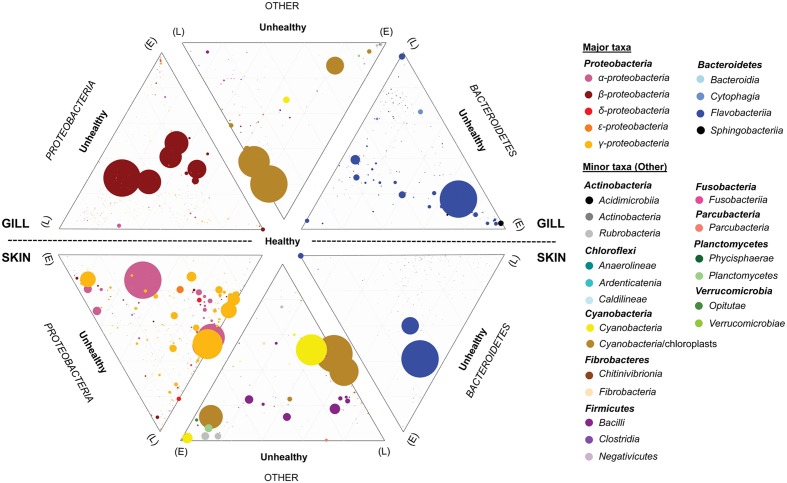
Ternary plots depicting the global bacterial taxonomic landscape of OTUs between the gills and skin of farmed healthy YTK and unhealthy YTK exhibiting early (E) and late (L) stage enteritis. Each plot represents the contribution of each OTU as assigned to the different taxonomic classes for the predominant phyla Proteobacteria and Bacteroidetes, and the other phyla. The size of the circles represents the relative abundance of the OTUs between all other taxa within the same plot. Those taxa with a percentage abundance of <0.1% in any one individual were omitted from the plots. Grids represent divisions of 0.2.

To establish the core bacterial assemblages of healthy YTK, from which inferences of health status can then be drawn, the diversity of the healthy skin and gills were first evaluated. Of the 764 healthy-YTK OTUs, a total of 652 were shared between skin and gills, where the majority (630) were not significantly different between these regions (**Figure 4A** and Supplementary Table S2). Of the 134 significantly different OTUs, 89 were more abundant in skin (where additional 44 were unique) and 45 were more abundant in gills (where additional 68 were unique) (**Figure 4A** and Supplementary Table S2). The most significantly abundant OTUs of the healthy skin belonged to α-proteobacteria (*Pelagibacter*, *Ascidiaceihabitans*, and *Roseovarius*), γ-proteobacteria (*Glaciecola* and the KI89A clade) and Cyanobacteria (*Synechococcus*) (**Figure 5**), while the most significantly abundant OTUs of the healthy gills belong to β-proteobacteria (unclassified SC-I-84, *Alcaligenes*, *Candidatus Glomeribacter*, *Ferrovum* spp. and Neisseriales) and Cyanobacteria/chloroplasts (**Figure 6**), features which are reflected in the global bacterial taxonomic landscape (**Figure 7**). In addition, OTU 6 (*Nitrosomonas* sp.), though not significantly different [*p*-value (corrected) = 0.052] had a notably higher abundance in gills overall (with an uncorrected *p*-value = 0.009) (**Figure 6** and Supplementary Table S2).

**FIGURE 8:**
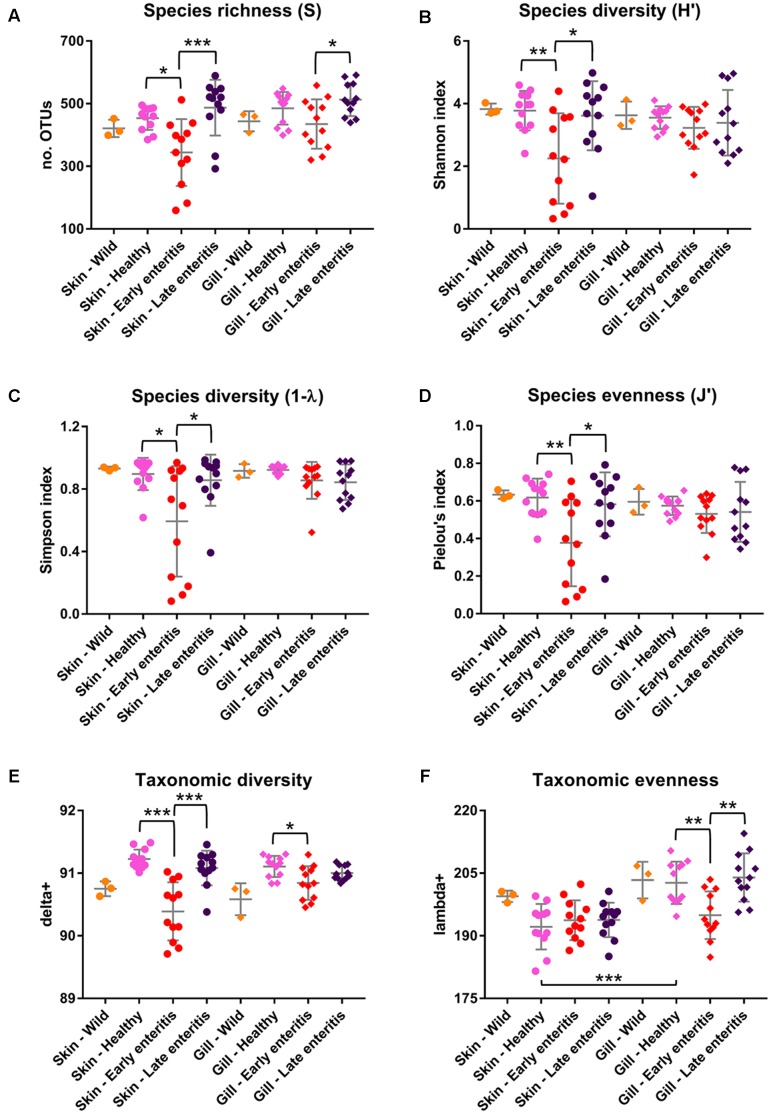
Measures of **(A)** Species richness, **(B)** Shannon diversity, **(C)** Simpson diversity, **(D)** Pielou’s evenness, **(E)** Taxonomic diversity (delta+), and **(F)** Taxonomic evenness (lambda+) of the skin and gill samples. Mean values and standard deviations are plotted for wild-caught YTK (orange), healthy farmed YTK (pink), and farmed YTK exhibiting early (red) and late (dark blue) stages of enteritis. Differences were evaluated between the skin and gills for each group using Welch’s *t*-test and between states of health for each region using one-way ANOVA with Tukey’s *post hoc* test. Asterisks denote levels of statistical significance between groups, with alpha set at 0.05.

The dominance of β-proteobacteria in the gills is reflected in the P:B ratio which shows that the mean number of Proteobacteria to Bacteroidetes is 10 while for the skin it is almost 2 and is highly significantly different between these groups (**Figure 3B**). However, this was not evident in the wild-caught fish (although only three individuals were sampled), where the mean P:B ratio of skin and gills was 14 and 4, respectively and was not significantly different. Species (OTU) richness was slightly higher in the healthy gills (mean 485, min–max 399–548) than the skin (mean 453, min–max 385–495), although was not significantly different (**Figure 8A**). Furthermore, fewer less dominant OTUs were apparent in the gill community compared to the skin (Supplementary Figure S6). The measures of species diversity (Shannon, Simpson, and Pielou’s evenness) were high and did not vary or significantly differ between the healthy skin and gills (**Figures 8B–D**). However, taxonomic diversity measured as TD, which reports on the relatedness between pairs of OTUs within each sample and their disparate range, shows that the taxonomic diversity of the skin was similar to the gills though was more even (skin mean delta+ 91.23 and mean lambda+ 192.2; gills mean delta+ 91.11 and mean lambda+ 202.7) (**Figures 8E,F** and Supplementary Figure S7). This indicates that the gills were dominated by OTUs of fewer distinct lineages, as was reflected in the high prevalence of mostly β-proteobacteria.

### The Skin-Associated Bacterial Assemblages across Different States of Health

To establish how the core skin-associated bacterial assemblages of healthy YTK might change in respect to changing health status (i.e., when the fish suffer from an underlying chronic disease which is unrelated to the skin and thus are likely to have an altered immune state and/or barrier system) the diversity of the skin between healthy and early and late stage enteritis groups was evaluated. As established above, there was a global significant difference and separation between health states (PERMANOVA pseudo-*F* = 6.7882, *p*-value = 0.0001; ANOSIM *R* = 0.411, *p*-value = 0.0001; Supplementary Table S1). Of particular note, the early enteritis group separated out further from both the healthy and late stage enteritis groups (ANOSIM: *R* = 0.645, *p*-value = 0.0001 and *R* = 0.337, *p*-value = 0.0008, respectively), while the healthy and late enteritis groups were more similar though significantly different (*R* = 0.246, *p*-value = 0.002), as clearly evident in **Figure 2**.

Of the 785 skin-associated farmed YTK OTUs, a total of 606 were shared between healthy and early and late stage enteritis fish, where 416 were not significantly different (**Figure 4B** and Supplementary Table S3). Of the 369 significantly different OTUs, 19 were unique to the healthy group, 12 to early enteritis, and 45 to late enteritis (**Figure 4B**). This is of particular merit, as while there were more significant differences in the early enteritis group in comparison to health and late stage enteritis, the late stage enteritis group did in fact have more unique OTUs. However, based on the heatmap, it is evident that both healthy and late stage enteritis groups were more similar, with the exception of one OTU (Cellvibrionales) that was more abundant in the late enteritis group. In stark contrast, there were dynamic changes in the abundance of OTUs from healthy to early stage enteritis fish. That is, the significantly abundant OTUs of healthy fish included: α-proteobacteria (*Ascidiaceihabitans*, *Roseovarius*, unclassified Rhodobacterales spp., and unclassified Rickettsiales sp.); β-proteobacteria (*Ferrovum* spp., unclassified Neisseriales sp., and *Azospira*); δ-proteobacteria (unclassified Desulfuromonadales sp.); γ-proteobacteria (*Alteromonas*, *Glaciecola*, *Idiomarina*, *Marinobacter*, *Pseudoalteromonas*, K189A clade, *Halomonas*, and *Photobacterium*); Bacteroidetes (unclassified Flavobacteriales spp.); Cyanobacteria (chloroplasts and *Synechococcus*); and Actinobacteria (*Corynebacterium*). Whereas in the early enteritis group the significantly abundant OTUs included: α-proteobacteria (*Loktanella*, *Marivita*, and *Planktomarina*); β-proteobacteria (*Simplicispira*); γ-proteobacteria (*Litoricola* and unclassified Oceanospirillales sp.); Cyanobacteria (chloroplast); Actinobacteria (*Candidatus Aquiluna*); and unclassified Planctomycetes sp. (**Figure 5**). These features are reflected in the global bacterial taxonomic landscape comparing all three conditions (**Figure 7**).

This observation is further strengthened when considering the surrounding seawater. Of the 15 most abundant OTUs of seawater collected adjacent to the early/late stage enteritis sea cage (seawater 2) (OTUs 9, 25, 105, 141, 232, 94, 123, 233, 6887, 288, 159, 47, 197, 98, and 130), which accounted for >72.5% of the total community abundance, five (OTUs 9, 25, 233, 98, and 130) as well as OTU 2530 and 43 were highly significantly abundant (with a large effect size) in the gills of the early enteritis group (**Figure 6**). OTUs 233, 98, 2530, and 43 were also significantly abundant (though with a lesser extent and effect size) in the skin of the early enteritis group (**Figure 5**). In contrast, of the 15 most abundant OTUs of seawater collected adjacent to the healthy sea cage (seawater 1) (OTUs 9, 25, 105, 141, 232, 94, 123, 233, 6887, 288, 159, 47, 197, 98, and 130), which accounted for >55.5% of the total community abundance, the majority were either of low abundance or absent from the healthy skin or gills (**Figures 5**, **6** and Supplementary Datasheet 1). Though OTU 25 and OTU 98, respectively belong to the γ-proteobacteria and Bacteroidetes, the predominant OTUs (9, 233, 130, 2530, and 43) belong to the α-proteobacteria (mostly of the order Rhodobacterales).

The number of Proteobacteria to Bacteroidetes is generally lower in the fish of the early enteritis group with a mean P:B ratio of <1, compared to both the healthy and late stage enteritis groups with each having a P:B ratio of 2, although is not considered significantly different (**Figure 3B**). Species richness was higher in the skin of late stage enteritis and healthy fish with a mean of 487 and 454 OTUs, respectively, compared to a mean of 344 OTUs for the early enteritis group being significantly different (**Figure 8A**). Also, as with species richness, the other measures of species diversity (Shannon, Simpson, and Pielou’s evenness) were all higher in both healthy and late stage enteritis groups which never differed significantly, as compared to the early enteritis group which always returned lower values for diversity and evenness, always being significantly different to both the healthy and late stage enteritis groups (**Figures 8B–D**). This was also reflected in the taxonomic diversity measures of average TD which also returned higher delta+ values which were highly significantly different to the early enteritis group. While the early enteritis group of YTK was much less taxonomically diverse (see the separation of the early enteritis group in **Figure 8E** and Supplementary Figure S7A), taxonomic evenness was equivalent between all three groups (**Figure 8F** and Supplementary Figure S7C), indicating that the reduced OTU diversity in this group did not necessarily lead to dominance of a few distinct lineages. The skin of fish from the early enteritis group also showed much higher dominance of fewer OTUs, where the bacterial assemblages of 6 out of the 12 fish were dominated by one OTU, either *Lutibacter* sp. (OTU 3) or *Polaribacter* sp. (OTU 8) (**Figure 5** and Supplementary Figure S6A). In contrast, most of the fish from the healthy and late stage enteritis groups had cumulative OTU dominance that was less pronounced (having gently rising curves which started lower on the plot), indicating less dominance, and greater levels of diversity and OTU evenness (Supplementary Figure S6A).

### The Gill-Associated Bacterial Assemblages across Different States of Health

There was also a global significant difference and separation between health states of the gills (PERMANOVA pseudo-*F* = 6.7525, *p*-value = 0.0001; ANOSIM *R* = 0.472, *p*-value = 0.0001; Supplementary Table S1). The early enteritis group, again, separated out further from both the healthy and late stage enteritis groups (ANOSIM: *R* = 0.584, *p*-value = 0.0001 and *R* = 0.473, *p*-value = 0.0001, respectively), while the healthy and late stage enteritis groups were more similar though significantly different (*R* = 0.425, *p*-value = 0.0001), as clearly evident in **Figure 2**.

Of the 817 gill-associated farmed YTK OTUs, a total of 666 were shared between healthy and early and late stage enteritis fish, where half (409) were not significantly different (**Figure 4C** and Supplementary Table S4). Of the 408 significantly different OTUs, 10 were unique to the healthy group, 33 to early enteritis, and 32 to late enteritis (**Figure 4C**). Based on the heatmap (**Figure 5**), it is again evident that both healthy and late stage enteritis groups were more similar, with the exception of four OTUs (unclassified Rhodobacterales sp., *Desulfobacter*, *Arcobacter*, and *Alteromonas*) that were more abundant in the late stage enteritis group. In contrast, like the skin there were notable changes in the abundances of OTUs from healthy to early enteritis fish. That is, the significantly abundant OTUs of healthy fish included: α-proteobacteria (*Ascidiaceihabitans*, *Roseovarius*, and unclassified Rhodobacterales spp.); β-proteobacteria (*Candidatus Glomeribacter* and *Polynucleobacter*); γ-proteobacteria (*Alteromonas* spp., *Glaciecola*, *Idiomarina*, *Marinobacter*, *Pseudoalteromonas*, *Cobetia*, *Halomonas*, *Psychrobacter*, and *Photobacterium*); Bacteroidetes (unclassified Flavobacteriales sp.); Cyanobacteria/chloroplasts; and Firmicutes (*Salimicrobium*). Whereas in the early enteritis group the significantly abundant OTUs included: α-proteobacteria (*Pelagibacter*, *Loktanella*, *Marivita*, and *Planktomarina*); β-proteobacteria (*Simplicispira*); γ-proteobacteria (unclassified Cellvibrionales sp., *Litoricola*, *Pseudospirillum*, and unclassified Oceanospirillales spp.); Bacteroidetes (NS5 Marine group and unclassified Sphingobacteriales); Cyanobacteria/chloroplasts; Actinobacteria (*Candidatus Aquiluna* and *Microcella*); and unclassified Planctomycetes sp. (**Figure 6**). These features are reflected in the global bacterial taxonomic landscape comparing all three health states (**Figure 7**).

Species richness was higher in the gills of late stage enteritis and healthy fish with a mean of 513 and 485 OTUs, respectively, compared to a mean of 435 OTUs for the early enteritis group, a finding being significantly different between the early and late stage enteritis groups (**Figure 8A**). Unlike in the skin, the other measures of species diversity (Shannon, Simpson, and Pielou’s evenness) as well as the P:B ratio were slightly higher in the healthy and late stage enteritis groups, compared to the early enteritis group, but never significantly different (**Figures 3B**, **8B–D**). However, the healthy and late stage enteritis groups returned higher values for both delta+ and lambda+ that were significantly different to the early enteritis group (**Figures 8E,F**). Interestingly, unlike the skin, the bacterial assemblages of the gills of healthy and late stage enteritis fish were more taxonomically uneven with a greater disparate range of delta+ and thus significantly higher values for lambda+, while still being taxonomically diverse, indicating the dominance of a few distinct lineages in these samples. In contrast, the early enteritis group of fish were slightly less taxonomically diverse with a more taxonomically even distribution of OTUs. Lastly, unlike in the skin, cumulative OTU dominance within the gill samples was less pronounced (Supplementary Figure S6B).

## Discussion

The bacterial assemblages associated with the outer surface mucosal barriers of healthy YTK were revealed. A core bacterial community was observed for both skin and gills, which was different to that of the surrounding environment, suggesting that YTK under healthy conditions can enrich and regulate host specific assemblages, which has been reported for other teleosts (Wang et al., 2010; Chiarello et al., 2015; Schmidt et al., 2015). Healthy YTK comprised mostly species of the Proteobacteria and Bacteroidetes, with a particular enrichment of β-proteobacteria in the gills and enrichment of Bacteroidetes and α- and γ-proteobacteria in the skin. Much of the published work concentrates on gut assemblages (see the review by Llewellyn et al., 2014), where within YTK it has recently been demonstrated that Proteobacteria (or Firmicutes) dominate the gut, depending on whether they originate from wild or cultivated systems, and likely reflects different contributions to the host (Ramirez and Romero, 2017). Of the few reports of the in-depth characterization of microbiota of the teleost skin and gills, Boutin et al. (2014) found that the skin of Brook Charr is also dominated by Proteobacteria and Bacteroidetes at similar proportions found in this work. However, comparisons between the skin microbiota of Striped Mullet, Red Snapper, Spotted Sea Trout, Sand Sea Trout, Pinfish, and Atlantic Croaker, reveals that while Proteobacteria is always the most dominant phylum of the skin, each fish comprises differing proportions of the other phyla Firmicutes, Bacteroidetes, Actinobacteria, and Cyanobacteria (Arias et al., 2013; Larsen et al., 2013).

In the present work, key groups of bacteria associated with healthy skin and gills were evident, with groups pertaining to the genera *Nitrosomonas* and *Ferrovum* largely enriched in the gills. Members of the Nitrosomonadales (like *Nitrosomonas*) are well-known ammonia oxidizers, where they convert ammonia to nitrite. In fish, the gills excrete most nitrogenous waste as ammonia, which in turn is actively used by these bacteria and converted to nitrite as has been documented for Common Carp and Zebrafish (van Kessel et al., 2016). This prevents the build-up of ammonia to toxic levels and also provides the bacteria with a rich nutrient supply (van Kessel et al., 2016). However, nitrite itself is also recognized as a toxic substance (Jensen, 1996). Studies have shown that nitrite can cause anoxia in fish and other aquatic animals (Lewis and Morris, 1986), as well as an increased susceptibility to bacterial diseases (Jensen, 1996). Nitrite also causes the condition of methemoglobinemia or brown blood disease, where the blood and gills of the fish darken due to lack of oxygen (Frances et al., 1998). However, with the presence of both ammonia-oxidizing and denitrifying microorganisms within fish gills, ammonia can be converted to nitrite by the former which is then converted to non-toxic dinitrogen gas by the latter, representing symbiosis between the fish host and these nitrogen cycling microorganisms (van Kessel et al., 2016). Ideal conditions for the proliferation of *Nitrosomonas* is suggested to occur at the interface between oxic environments with high oxygen concentrations and anoxic environments of high ammonium concentrations, as these organisms require oxygen to form nitrite (Kirchman, 2012). Thus the gills present an ideal organ for the enrichment of these organisms, which also seems to be important symbionts in YTK.

Of particular interest here was the predominance of other β-proteobacterial OTUs within the gills representing the genus *Ferrovum* who, as members of the “iron bacteria,” obtain their energy by catalyzing the oxidation of ferrous iron (Hallberg et al., 2006). Curiously, members of this genus such as *Ferrovum myxofaciens* (which was the closest hit for OTU 18) are extreme acidophiles with an optimum pH of 3.0 (Kimura et al., 2011). Considering that marine waters have a pH of 8.3–8.4 and a half-life of ferrous iron is only ∼2 min (Millero et al., 1987), their occurrence here is particularly unusual given that the pH of the surfaces of the gills is typically alkaline (pH ∼8.0) (Lewis and Morris, 1986). Moreover, whilst iron oxidizing bacteria are widely distributed in environmental systems (particularly in aquatic environments) (Hedrich et al., 2011), they do not appear to be typically associated with eukaryotic hosts and their co-occurrence here with organisms like *Nitrosomonas* seems counterintuitive given that ammonium oxidation would most likely be inhibited at any pH below 7. That said, earlier reports on fish diseases have, however, documented the association of certain iron bacteria like *Leptothrix ochracea* Kütz and other unidentified species in association with farmed Trout and Common Carp where they cover the gill lamellae and inhibit respiration by forming insoluble iron deposits (Schäperclaus, 1992; Svobodová et al., 1993; Slaninova et al., 2014). Furthermore, a more recent report has also indicated the occurrence of other iron-oxidizing bacteria (like Zetaproteobacteria) as epibionts within the gills of deep-sea shrimp (Jan et al., 2014). Since iron uptake is regulated through the gills where it is actively sequestered as part of the host’s natural defenses and for use in various cellular processes, it also provides an ideal source for opportunists (Collins, 2008; Bury et al., 2012). Interestingly, genomic reconstructions of various *Ferrovum* strains reveal that these organisms may also have the capacity to use ammonium, urea and nitrate as nitrogen sources (Hua et al., 2015; Ullrich et al., 2016). Given this and the widely variable physiological optima of the iron bacteria as a whole, where species may also be moderately acidophilic or even neutrophilic (Hedrich et al., 2011), one might consider the possibility that this bacterium may have the capacity to colonize the gill tissues, perhaps in localized areas of low pH arising from acid production from fermentative bacteria where, like *Nitrosomonas*, they may utilize the excreted nitrogenous wastes from the host. However, given that only relatively short OTU sequence reads were obtained here, the occurrence of this bacterium and its relevance in YTK (and perhaps fish more broadly) requires further elucidation.

Changes in the bacterial assemblages associated with the outer surface mucosal barriers of YTK during early and late stages of chronic lymphocytic enteritis were also revealed. Features accompanying this and reflecting changes in global health and fitness of these animals included a general reduction in body size and condition as well as changes in the barrier system with a loss of skin thickness and acidic mucus producing goblet cells, indicating important structural and/or physiological changes. This was marked by an inferred shift in the global bacterial assemblages, especially between healthy and early stages of enteritis, where there was an overall loss in diversity with enrichment of specific groups within the α-, γ-proteobacteria, and Actinobacteria, suggesting a shift in the global outer-surface mucosal assemblages with the onset of disease. This was particularly evident in the gills where many of these taxa (especially α-proteobacteria of the order Rhodobacterales) were also found in relatively high abundances within the surrounding seawater, a feature not apparent for the healthy fish. A further shift in the community between the early and later stages of enteritis was observed, with a reduction in the abundance of these early stage enriched taxa and a concomitant increase in overall diversity similar to the healthy fish. Studies on other teleosts have also noted a change in the skin and gill microbiota in response to underlying stressors (Roberts and Powell, 2005; Hess et al., 2015; Mohammed and Arias, 2015). Interestingly, the α-subdivision of the Proteobacteria which inhabit a wide range of ecosystems and have become adapted to eukaryotic hosts where they form both extra- and intracellular associations, has evolved an array of chronic infection strategies (Batut et al., 2004). Their emergence with the skin and gill mucosa during early stages of infection here may thus represent favorable conditions brought about by the loss of barrier function (and potentially changes in pH of the mucosal surfaces due to loss of local acidic producing goblet cells) in the weakened host. Although it is difficult to explain why the host reverts back to a composition similar to the healthy individuals, it is tempting to speculate that this represents further subsequent immunological changes from early to late stages of enteritis. Furthermore, this also suggests that common mucosal responses exist or that there is direct link between the mucosa of the gut and the external surfaces (Xu et al., 2013, 2016), where responses are body-wide irrespective that the symptoms of the disease are localized to the gut, which requires further elucidation. This is particularly pertinent given an earlier report of the body-wide mucosal responses in the skin and gills of Atlantic Salmon and Brown Trout (Roberts and Powell, 2005).

Specific changes in the microbiota of the skin and gills between early and late stages of enteritis were also apparent, though not as prominent when compared to healthy fish. In particular, taxa belonging to the Desulfobacterales (namely *Desulfobacter*) and Campylobacterales (*Arcobacter*), among others, were enriched during the late stages of enteritis. As a group of strictly anaerobic bacteria that utilize sulfur as their primary energy source where they reduce sulfate to sulfide and occur in a range of ecosystems including aquatic habitats and the host (GI tract) mucosa (Purdy et al., 2002; Fera et al., 2004; Croix et al., 2011), the enrichment of *Desulfobacter* during late stages of enteritis may indicate an even more profound change in the host mucosa. More specifically, though naturally occurring sulfur compounds exist in the fish mucus as sulfomucins (Wilson and Castro, 2010), further deterioration in the condition of the animal and its associated epithelial barriers (as reflected by the substantial decrease in skin thickness during disease) may lead to further enrichment of sulfur as well as impaired gas exchange (creating anoxic conditions), thereby providing a suitable environment for these organisms. In addition, the occurrence of aerotolerant organisms like *Arcobacter* which as a group of largely pathogenic organisms whose diversity include species which can also utilize sulfur (Wirsen et al., 2002; Collado and Figueras, 2011), may highlight the changes in the underlying conditions and the emergence of perhaps secondary pathogens in an already weakened host.

The wealth of information generated here can be used to propose some key markers for a change in health state of farmed fish. While the use of specific bacterial groups has been proposed as useful biomarkers in previous work, such as β-proteobacteria (*Flavobacterium*, *Duganella*, and/or *Undibacterium*) for monitoring stress to acidic conditions in Amazonian Tambaqui fish (Sylvain et al., 2016), often such examples do not represent indigenous microbiota of all fish, as is the case here with YTK. Thus, more global changes to proportions of the most predominant phyla have been suggested, like the Firmicutes:Bacteroidetes (F:B) ratio which was originally proposed for human and mammal systems (Ley et al., 2005, 2006; Turnbaugh et al., 2006) and where an increased F:B ratio has been associated with obesity, diabetes, irritable bowel syndrome, and cardiovascular disease in humans (Payne et al., 2011; Brown et al., 2012; Guinane and Cotter, 2013; Yang et al., 2015). Although, more recently in fish, this same approach was used to assess stress in Amazonian Tambaqui (Sylvain et al., 2016) and growth of Common Carp (Li et al., 2013). However, Firmicutes and Bacteroidetes are not always the predominant phyla of the fish mucosa, where in fact, Proteobacteria are often much more dominant (Boutin et al., 2014; Llewellyn et al., 2014; Ramirez and Romero, 2017). Thus, we propose here the P:B ratio instead. While we did not see a major difference in the P:B ratio between health states, larger discrepancies between individual fish with early stages of enteritis were observed, particularly in the skin and may represent an early indicator of an altered microbiome state. In addition, a difference between the skin of wild-caught and farmed YTK was apparent which, as similarly reported for the gut of this species (Ramirez and Romero, 2017) may provide a potential global biomarker of cultivation and warrants further investigation. The enrichment of specific groups of bacteria under differing stages of health, particularly at early stages of enteritis where diagnosis based on symptomatic features remains difficult, may also be useful indicators. As described above, a number of taxa including, among others, representatives of the α-proteobacteria (*Pelagibacter*, *Loktanella*, *Marivita*, and *Planktomarina*), γ-proteobacteria (*Litoricola*), and Actinobacteria (*Microcella* and *Candidatus Aquiluna*) may be useful in this regard.

## Conclusion

Our results indicate that gut health status is an important factor which defines the skin and gill bacterial assemblages of fish. In healthy fish, it is apparent that these niches comprise similar core communities though with enrichment of certain groups, particularly within the gills. This suggests that these niches may comprise additional resources which are utilized by these groups as relevant symbionts or opportunists. However, disease (enteritis) onset was marked by an overall loss in diversity with the emergence of specific community members, particular during the acute stages, likely reflecting changes in the immune states and the connectivity of these barrier systems. This study represents the first to investigate whether the onset of chronic gut enteritis is reflected in the microbiota of the outer-surface mucosal barriers of fish, where such knowledge can be used to detect early signs of a shift in health. This is particularly relevant as this is achieved non-invasively, whereby management of such conditions can then be implemented at the early stages of disease. To this end, future research should be directed toward understanding the underlying functional contribution and connectivity of these mucosal microbiomes (across the skin, gills, and gut) and their interplay with the host during changes in health.

## Ethics Statement

This study was exempt from ethics approval as the animals were privately owned and were sampled under the auspices of a commercial aquaculture enterprise from temperate waters of southern Australia according to industry best practice veterinary care.

## Author Contributions

AO conceived the initial project idea. AO, SC, and MW-O designed the approach to be used. ML provided clinical assessments and defined the sample populations. AO and SC collected the samples. FS performed histology and provided interpretation of the pathology of the specimens. SC and TL generated the laboratory data. MW-O provided guidance on the analysis of the data. MW-O, TL, and AO collated, analyzed, and interpreted the data. MB provided guidance with animal handling and assisted with the collection and provision of trial samples. DS and JQ provided overall project guidance and support. AO, TL, and MW-O wrote the paper. All authors provided comment on the manuscript prior to submission.

## Conflict of Interest Statement

The authors declare that the research was conducted in the absence of any commercial or financial relationships that could be construed as a potential conflict of interest.

## Footnotes

1 http://bioinformatics.psb.ugent.be/webtools/Venn/
