# Supplementary material for: The Inner Workings of the Outer Surface: Skin and Gill Microbiota as Indicators of Changing Gut Health in Yellowtail Kingfish
Source: Front Microbiol. 2018 Jan 15;8:2664. doi: 10.3389/fmicb.2017.02664 (PMC5775239; doi:10.3389/fmicb.2017.02664)
Supplement: Supplementary file 6 [file Data_Sheet_2.PDF]

frontiers

Supplementary Material

The Inner Workings of the Outer Surface: Skin and Gill Microbiota as Indicators of Changing Gut Health in Yellowtail Kingfish

Thibault P.R.A. Legrand[1,2†], Sarah R. Catalano[1†], Melissa L. Wos-Oxley[3,4†], Fran Stephens[5], Matt Landos[6], Matthew S. Bansemer[1], David A.J. Stone[1,2], Jian G. Qin[2] and Andrew P.A. Oxley[1*]

*** Correspondence:** Dr Andrew Oxley: Andrew.Oxley@sa.gov.au

1 Supplementary Tables

Supplementary Datasheet 1 (XLSX) Relative percent abundance, phylogenetic assignment and nucleotide assignment of all 930 OTUs across the 80 samples, following Illumina-based amplicon deep-sequencing.

Table S1 (XLSX) PERMANOVA and ANOSIM results tables.

Table S2 (XLSX) Differential abundance of OTUs between healthy skin and healthy gills. The Welsh's t-test with correction for p-values was performed using the Benjamini-Hochberg multiple comparison test, where alpha was set at 0.05. Of the 764 skin-gill-associated OTUs, 134 returned p-values <0.05 (bolded) and of these 15 have an effect size (using the "difference between proportions" option) <1.0 (underlined).

Table S3 (XLSX) Differential abundance of OTUs between health states (Healthy, Early enteritis, Late enteritis) within the skin microbiome. The Kruskal-Wallis test with the Games-Howell post hoc test was applied and correction for p-values was performed using the Benjamini-Hochberg multiple comparison test, where alpha was set at 0.05. Of the 785 skin-associated OTUs, 369 returned p-values <0.05 (bolded) and of these 32 have an effect size >0.5 (underlined).

Table S4 (XLSX) Differential abundance of OTUs between health states (Healthy, Early enteritis, Late enteritis) within the gill microbiome. The Kruskal-Wallis test with the Games-Howell post hoc test was applied and correction for p-values was performed using the Benjamini-Hochberg multiple comparison test, where alpha was set at 0.05. Of the 817 gill-associated OTUs, 408 returned p-values <0.05 (bolded) and of these 80 have an effect size >0.5 (underlined).

2 Supplementary Figures

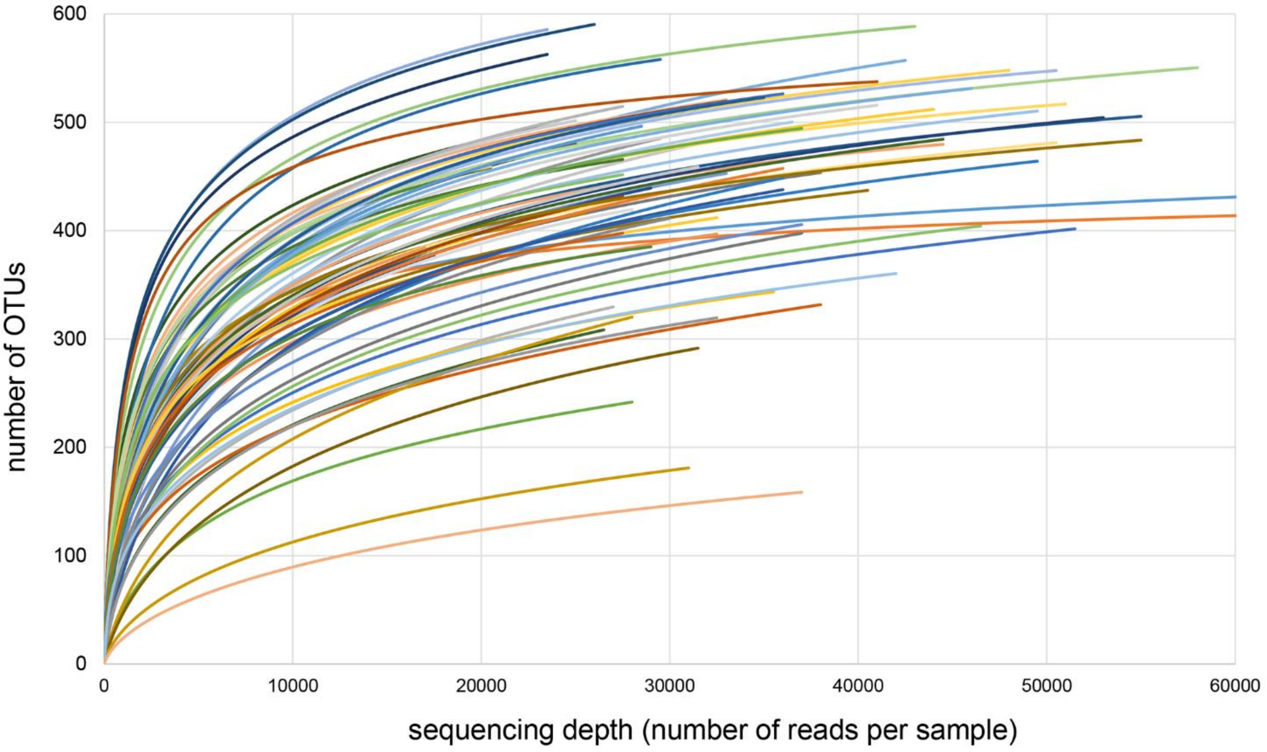

Figure S1 Rarefaction curves depicting the number of resolved OTUs against sequencing depth of the 80 samples (39 gill swabs, 39 skin swabs and 2 seawater samples).

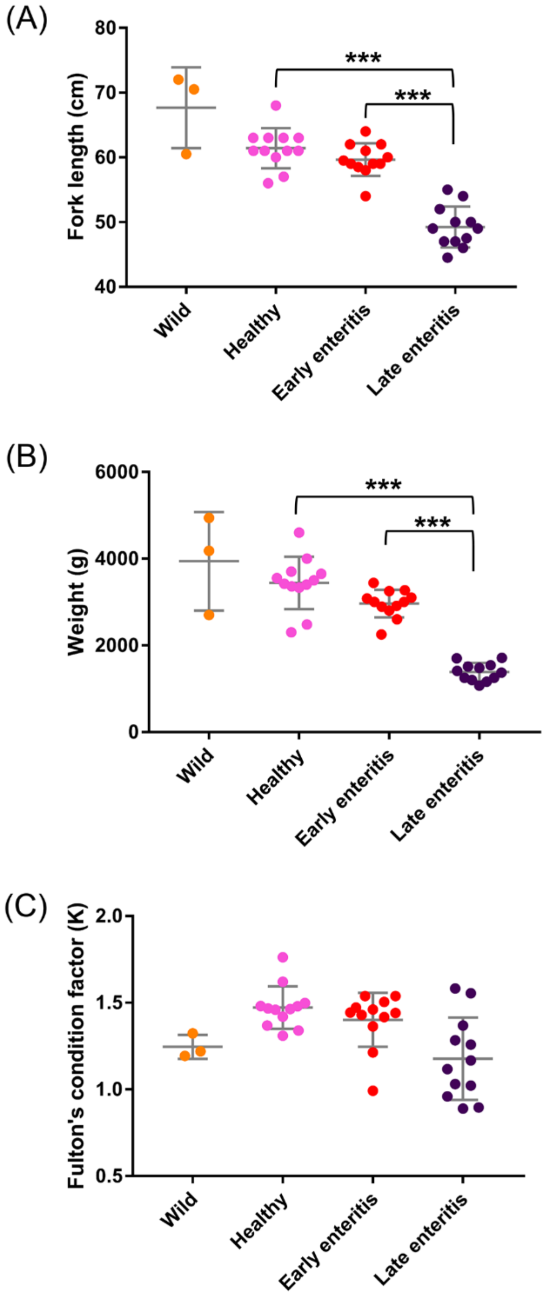

Figure S2 Growth characteristics of YTK where plots represent measurements of (A) fork length, (B) weight and (C) Fulton's condition factor of wild-caught YTK (orange), healthy farmed YTK (pink), and farmed YTK with early (red) and late stage enteritis (dark blue). The mean and standard deviation are presented for each group. Differences between groups were evaluated using one-way ANOVA with Tukey's post hoc test with the levels of statistical significance between groups (excluding wild-caught YTK) denoted by asterisks, with alpha set at 0.01.

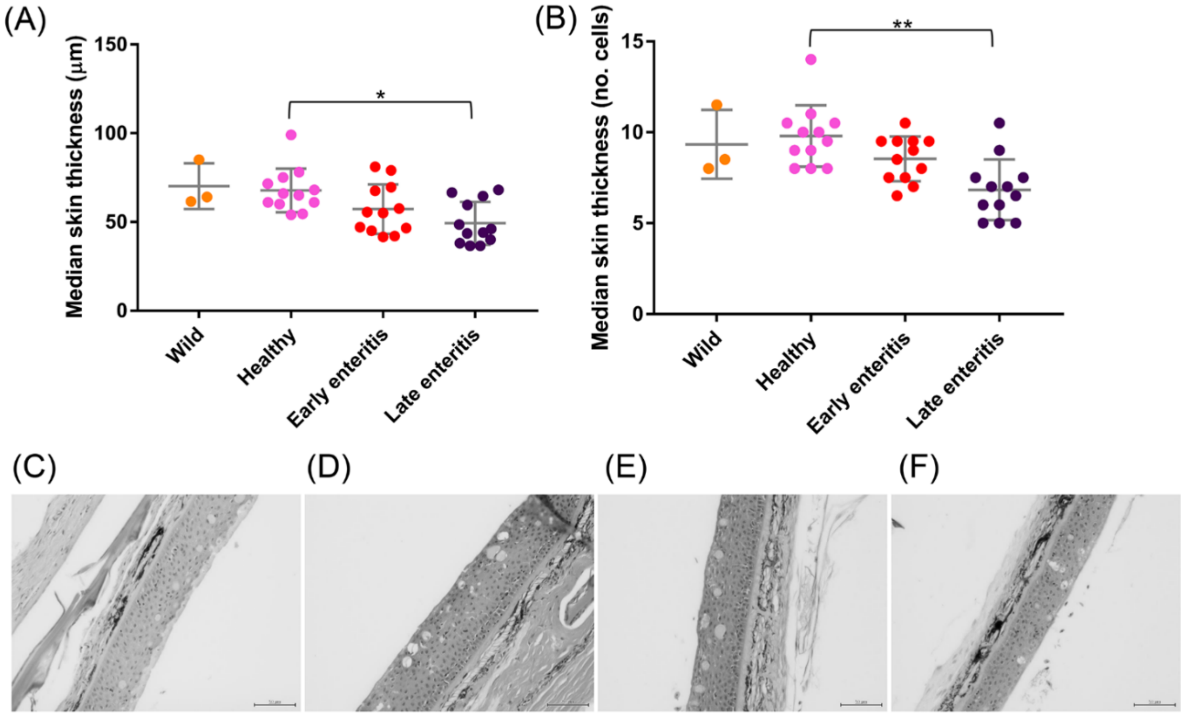

Figure S3 Median skin thickness of wild-caught YTK, healthy farmed YTK and farmed YTK with early and late stage enteritis as revealed from HE sections where plots represent (A) thickness in µm and (B) number of cells thick. Within each plot the colored dots represent wild-caught YTK (orange), healthy farmed YTK (pink) and farmed YTK with early (red) and late (dark blue) stages of enteritis. The mean and standard deviation are presented for each group. Differences between groups were evaluated using one-way ANOVA with Tukey's post hoc test with the levels of statistical significance between groups (excluding wild-caught YTK) denoted by asterisks, with alpha set at 0.01. Representative HE sections from (C) wild, (D) healthy, (E) early enteritis and (F) late enteritis YTK depict mean skin thickness (scale bar = 50 µm).

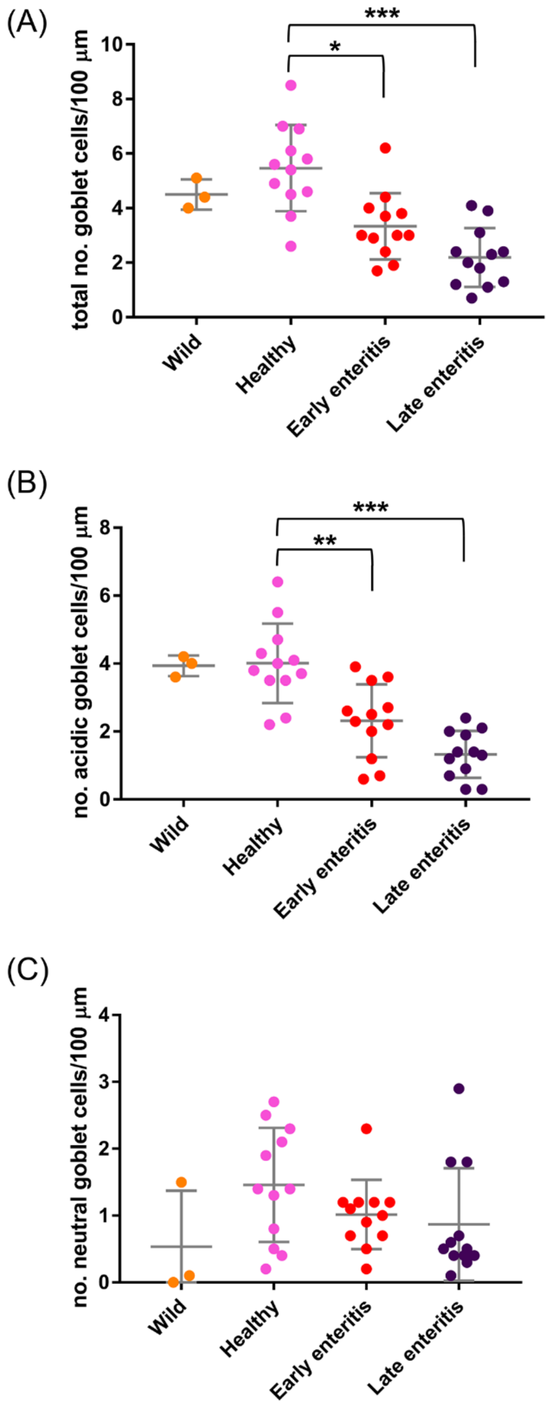

Figure S4 Mean goblet cell counts from the skin of wild-caught YTK, and farmed YTK of varying health status using PAS-AB stained tissues sections where each plot represents (A) total number of goblet cells, (B) number of acidic goblet cells, and (C) number of unstained (neutral) goblet cells. Counts were obtained from five 100 µm sections from each fish with the mean and standard deviation presented for each group. Within each plot the colored dots represent wild-caught YTK (orange), healthy farmed YTK (pink) and farmed YTK with early (red) and late (dark blue) stages of enteritis. Differences between groups were evaluated using one-way ANOVA with Tukey's post hoc test with the levels of statistical significance between groups (excluding wild-caught YTK) denoted by asterisks, with alpha set at 0.01.

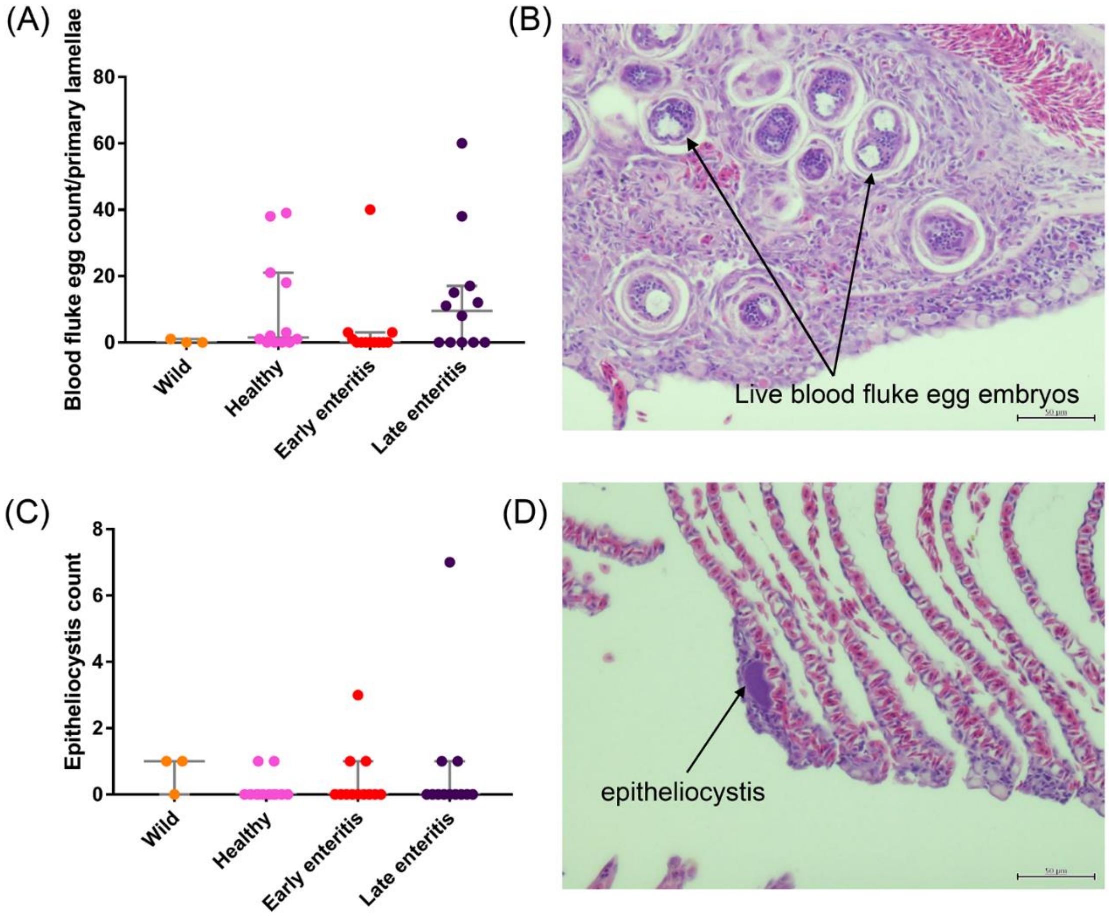

Figure S5 Total counts of (A) viable blood fluke eggs and (C) epitheliocystis, as obtained from HE stained sections of gills (B, D) from wild-caught YTK, healthy farmed YTK and farmed YTK with early and late stage enteritis. Within each plot the colored dots represent wild-caught YTK (orange), healthy farmed YTK (pink) and farmed YTK with early (red) and late (dark blue) stages of enteritis.

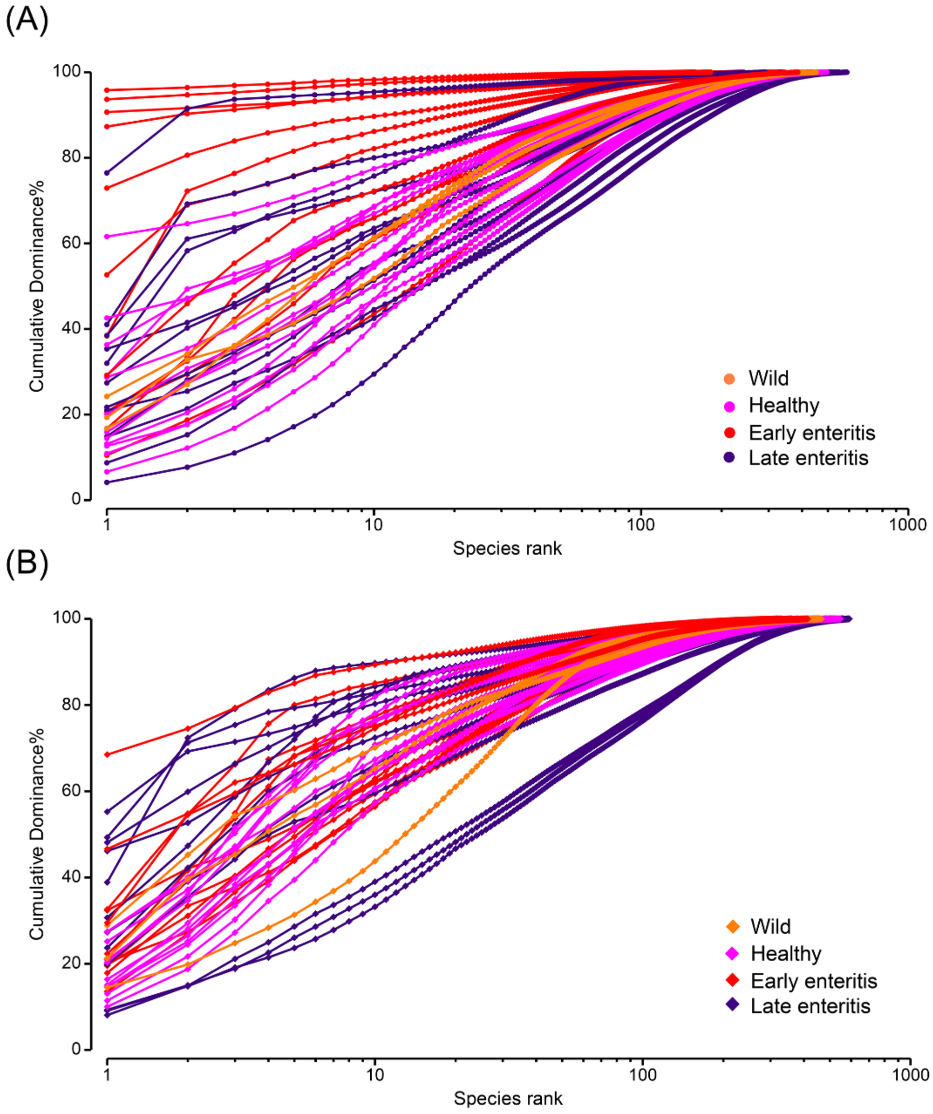

Figure S6 *k*-dominance plots presenting cumulative ranked abundances plotted against species rank depicting the diversity and dominance of bacterial OTUs, comparing wild-caught YTK (orange), healthy farmed YTK (pink) and farmed YTK exhibiting early (red) and late (dark blue) stages of enteritis from both (A) skin and (B) gills.

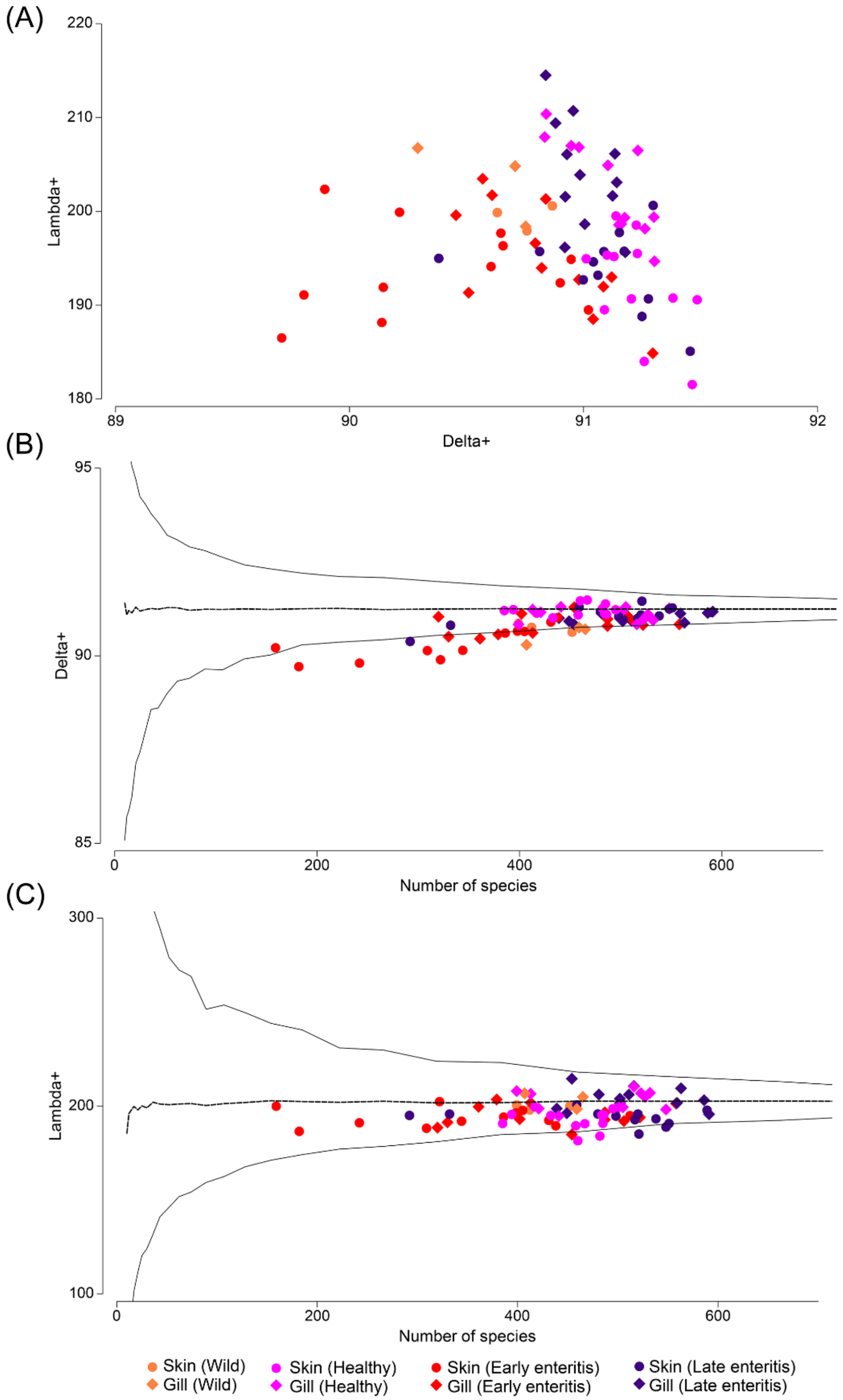

Figure S7 Taxonomic distinctness (TD) measures of bacterial communities from the skin and gills of wild-caught YTK, healthy farmed YTK and farmed YTK with early and late stage enteritis. Plots chart the average taxonomic distinctness (avTD, delta+) as a function of variation in (A) taxonomic distinctness (varTD, lambda+) and (B) avTD, and (C) varTD plotted against species (OTU) richness.
